# Electrochemical promotion of catalysis over Pd nanoparticles for CO_2_ reduction†
†Electronic supplementary information (ESI) available. See DOI: 10.1039/c6sc04966d



**DOI:** 10.1039/c6sc04966d

**Published:** 2017-01-03

**Authors:** Fan Cai, Dunfeng Gao, Hu Zhou, Guoxiong Wang, Ting He, Huimin Gong, Shu Miao, Fan Yang, Jianguo Wang, Xinhe Bao

**Affiliations:** a State Key Laboratory of Catalysis , CAS Center for Excellence in Nanoscience , Dalian Institute of Chemical Physics , Chinese Academy of Sciences , 116023 , Dalian , China . Email: wanggx@dicp.ac.cn ; Email: xhbao@dicp.ac.cn; b College of Chemical Engineering , Zhejiang University of Technology , 310032 , Hangzhou , China; c University of Chinese Academy of Sciences , 100039 , Beijing , China

## Abstract

Electrochemical promotion of catalysis (EPOC) has been shown to accelerate the rate of many heterogeneous catalytic reactions; however, it has rarely been reported in low-temperature aqueous electrochemical reactions. Herein, we report a significant EPOC effect for the CO_2_ reduction to generate formate over Pd nanoparticles (NPs) in a 1 M KHCO_3_ aqueous solution. By applying a negative potential over differently-sized Pd NPs, the rate of formate production is greatly improved as compared to that at an open-circuit voltage, with a rate enhancement ratio ranging from 10 to 143. The thermocatalytic and electrocatalytic reduction of CO_2_ compete with each other and are promoted by the applied negative potential and H_2_ in the feeds, respectively. Inspired by the EPOC effect, a composite electrode containing Pd/C and Pt/C catalysts on different sides of a carbon paper was constructed for catalyzing the CO_2_ reduction without adding H_2_ to the feeds. Water electrolysis over Pt NPs generates H_2_, which then effectively promotes formate production over Pd NPs.

## Abstract 2

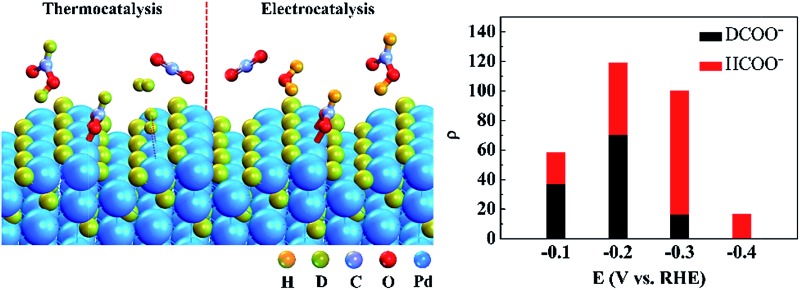
Electrochemical promotion of catalysis was observed over Pd nanoparticles with a significant rate enhancement ratio (*ρ*) for catalyzing CO_2_ reduction to produce formate in 1 M KHCO_3_ solution at ambient temperature.

## Introduction

Electrochemical promotion of catalysis (EPOC), discovered by M. Stoukides and C. Vayenas in the 1980s,^1,2^ has been widely investigated in more than 100 heterogeneous catalytic reactions^3–6^ on either metal or metal oxide surfaces, which are interfaced with a solid^7^ or an aqueous electrolyte solution.^8–14^ By applying an electrical current between the working electrode, coated with catalyst, and the counter electrode, the electronic properties of the supported catalyst can be tuned, accompanied by the alteration in the adsorption strength of the reactants, and in some cases, a significant enhancement in the catalytic performance can be observed. To date, only a few reports have demonstrated the EPOC effect in an aqueous electrolyte solution at ambient temperature for reactions such as the H_2_ oxidation,^8,9^ hydrocarbon isomerization,^10,11^ CO oxidation,^12,13^ and hydrazine oxidation.^14^ Herein, we report that the EPOC effect can also be observed for electrochemical reduction reactions such as the reduction of CO_2_ in an aqueous electrolyte solution at ambient temperature.

The reduction of carbon dioxide to produce formic acid is an attractive route to store renewable electricity and an important strategy for the utilization of carbon cycle.^7,15–19^ However, CO_2_ is thermodynamically stable and notoriously unreactive; therefore, high reaction temperatures^7,20–23^ and high overpotentials^24–27^ are usually essential to activate and transform CO_2_ during thermocatalysis and electrocatalysis. Herein, we report a significant EPOC effect for the CO_2_ reduction to produce formate over Pd nanoparticles (NPs) in a 1 M KHCO_3_ aqueous solution at ambient temperature. Thermocatalytic and electrocatalytic reduction of CO_2_ over Pd nanoparticles (NPs) occur simultaneously and compete with each other, which are promoted by the applied negative potential and H_2_ in the feeds, respectively. The shared reaction intermediate, namely HCOO*, is formed over the Pd NPs and is proposed as the origin of the EPOC effect during the thermocatalytic and electrocatalytic reduction of CO_2_. Inspired by the EPOC effect, a Pd/C–Pt/C composite electrode was constructed for the CO_2_ reduction, such that the addition of H_2_ to the feeds could be avoided. H_2_ generated from the electrolysis of water over Pt NPs effectively promotes formate production over Pd NPs.

## Results and discussion

Carbon-supported Pd NPs were prepared using sodium citrate as a stabilizing agent and NaBH_4_ as a reducing agent.^28^ Each sample was named on the basis of the average size of Pd NPs present in it, such as 3.7 nm Pd indicates that Pd NPs in the Pd/C catalyst have an average particle size of 3.7 nm (Fig. S1†). The catalyst ink, containing Pd/C and Nafion ionomer, was deposited on a piece of carbon paper (Toray TGP-H-060) with a microporous layer and dried to serve as a porous electrode for CO_2_ reduction in a 20% H_2_/CO_2_-saturated 1 M KHCO_3_ solution. To quantify the EPOC effect, the rate enhancement ratio was defined as follows:^3^

1 *ρ* = *r*/*r*_0_

where *r* and *r*_0_ are the rates of the promoted (by applying negative potentials) and unpromoted (at open-circuit voltage, OCV) reactions, respectively. In this study, all *r* values measured under different atmospheres or over different electrodes are calculated by the following equation:2
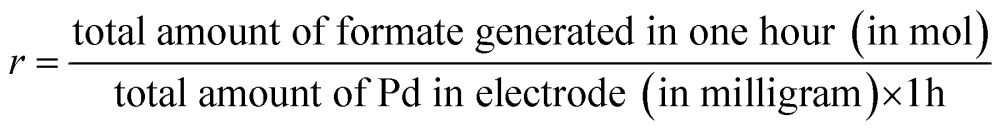



**Fig. 1:**
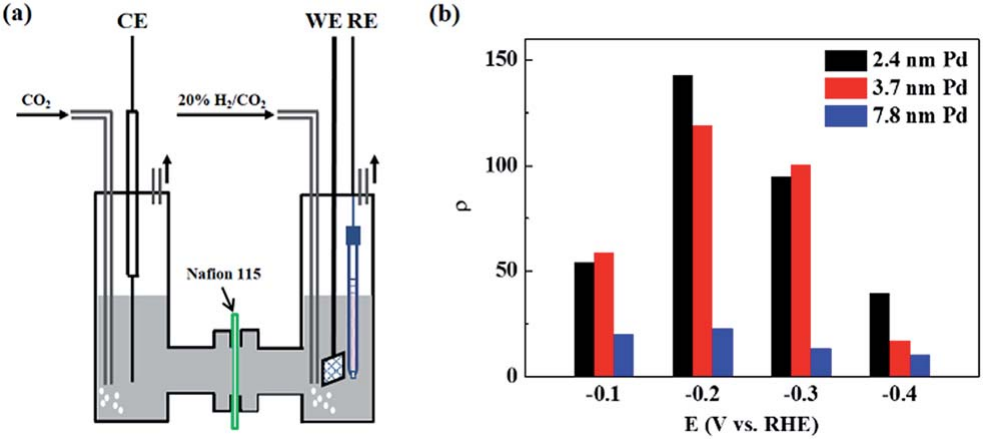
(a) Schematic of the H-cell for CO_2_ reduction. (b) Rate enhancement ratios for formate production at different negative potentials compared to those at OCV over differently-sized Pd NPs in 20% H_2_/CO_2_-saturated 1 M KHCO_3_ solution.

CO_2_ reduction experiments were conducted in an H-cell, as shown in Fig. 1a. The porous electrode coated with Pd/C catalyst was immersed in a 1 M KHCO_3_ aqueous solution, and 20% H_2_/CO_2_ was fed into the cathode chamber for the CO_2_ reduction reaction at OCV and different negative potentials. The maximum temperature increment of the electrolyte solution was 0.8 °C during the constant-potential electrolysis for 1 h, and the effect of temperature on the formate production rate at OCV and different negative potentials can be ignored. Fig. 1b shows the rate enhancement ratio for the formate production at different negative potentials compared to that at OCV over differently-sized Pd NPs. There are volcano-like curves for the value of *ρ* over differently-sized Pd NPs within the studied potential range. The value of *ρ* is 54 over 2.4 nm Pd at –0.1 V and reaches the maximum value of 143 at –0.2 V. Further negatively shifting the potential to –0.3 V and –0.4 V would decrease the rate enhancement ratio to 95 and 39, respectively. The ratio over 3.7 nm Pd increases from 58 to 119 when the potential is shifted from –0.1 V to –0.2 V and drops to 100 and 17 at –0.3 V and –0.4 V, respectively. The ratio over 7.8 nm Pd is obviously smaller than that over 2.4 nm Pd and 3.7 nm Pd, and the maximum ratio over 7.8 nm Pd is 23 at –0.2 V. The reaction of adsorbed hydrogen on Pd hydride surface with CO_2_ to form adsorbed HCOO* is considered as the rate-determining step for CO_2_ reduction.^16^ Upon negatively shifting the potential from –0.1 V to –0.4 V, the hydrogen adsorption strength on the Pd hydride surface is weakened due to a favored hydrogen evolution reaction. Therefore, the optimum adsorption strength for surface-adsorbed hydrogen to react with CO_2_ occurs at –0.2 V, resulting in the highest *ρ* value. Since small-sized Pd NPs prefer to adsorb more hydrogen over coordinatively unsaturated sites,^29^ the *ρ* value is much higher over 2.4 nm Pd as compared to that over 3.7 nm Pd and 7.8 nm Pd at –0.4 V.

**Fig. 2:**
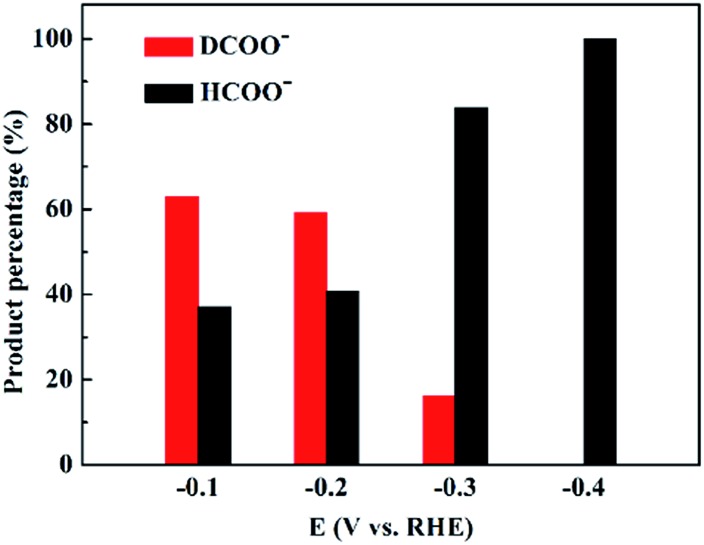
HCOO^–^ and DCOO^–^ percentages for the CO_2_ reduction over 3.7 nm Pd at different negative potentials in 20% D_2_/CO_2_-saturated 1 M KHCO_3_ solution.

To investigate the origin of the EPOC effect during CO_2_ reduction, an isotope-labeling experiment was conducted by replacing 20% H_2_/CO_2_ with 20% D_2_/CO_2_, and the products were analyzed by nuclear magnetic resonance (NMR) spectroscopy. PdD_*x*_ could also be generated under D_2_ atmosphere, and the properties of PdD_*x*_ were close to those of PdH_*x*_; thus, it was suggested that the electrochemical measurements under 20% D_2_/CO_2_ atmosphere were comparable to those under 20% H_2_/CO_2_ atmosphere.^30–32^ HCOO^–^ and DCOO^–^ were quantified by ^1^H-NMR and ^2^H-NMR spectra, respectively. The amount of D_2_O and HDO in the electrolyte solution after constant-potential electrolysis at –0.2 V for 1 h was also quantified by ^2^H-NMR spectra, which is 0.028%, whereas the natural abundance of D is about 0.015%.^33^ Therefore, non-electrochemical exchange between adsorbed D and H^+^ could be ignored,^34^ and DCOO^–^ and HCOO^–^ were considered to be produced from the CO_2_ + D_2_ thermocatalytic reaction and CO_2_ electrocatalytic reaction, respectively. Fig. 2 shows the percentage of HCOO^–^ and DCOO^–^ formed over 3.7 nm Pd at different negative potentials. The percentage of DCOO^–^ is higher than that of HCOO^–^ at –0.1 V and –0.2 V, which sharply decreases at –0.3 V and reaches below the detection limit at –0.4 V. Thus, the thermocatalytic and electrocatalytic reduction of CO_2_ occur simultaneously and compete with each other when applying negative potentials over Pd NPs.

**Fig. 3:**
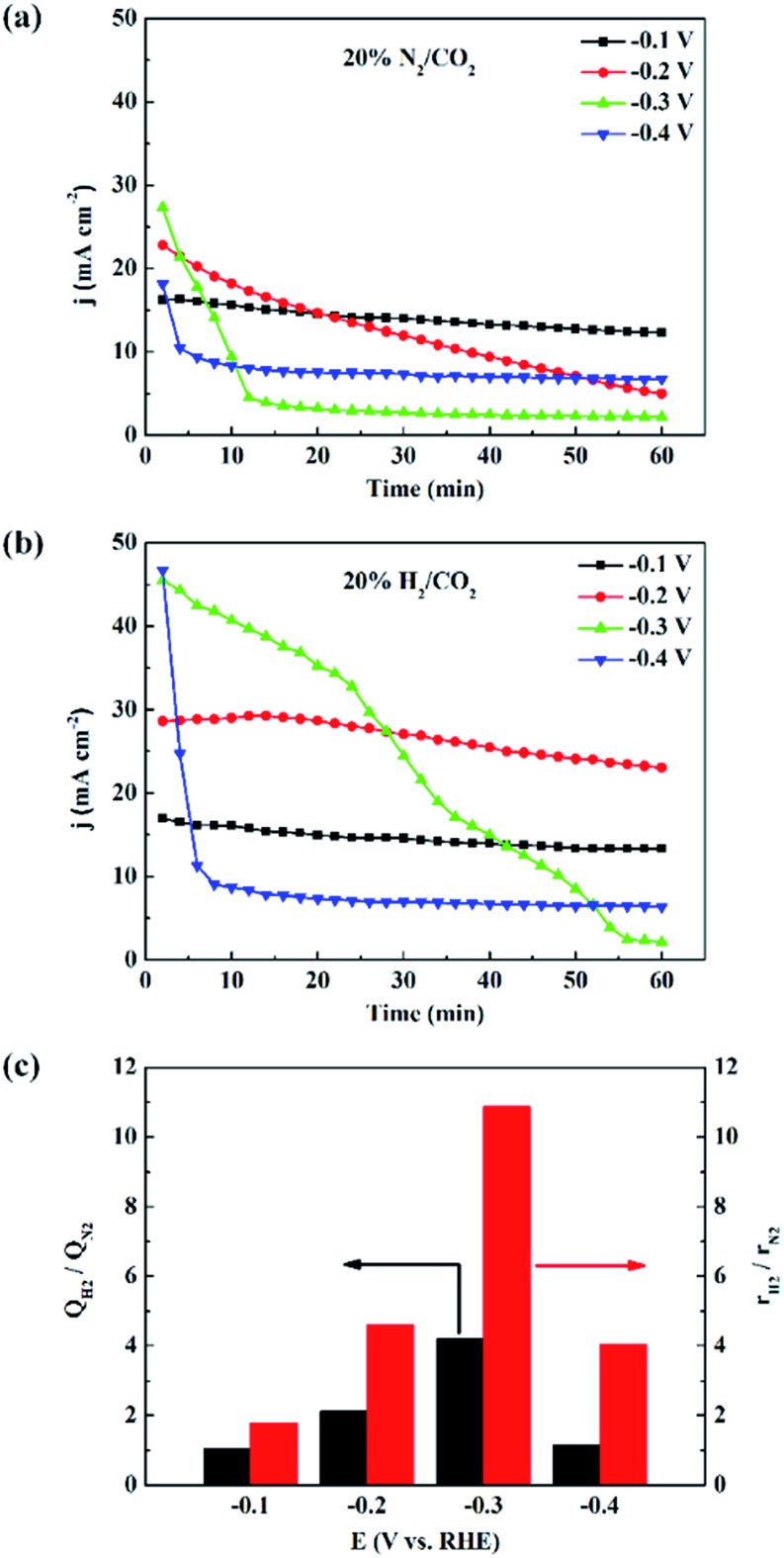
Chronoamperometry curves of 3.7 nm Pd in 20% N_2_/CO_2_-saturated (a) and 20% H_2_/CO_2_-saturated (b) 1 M KHCO_3_ solution. (c) Enhancement of electric charge (*Q*_H_2__/*Q*_N_2__) ratio and formate production rate ratio (*r*_H_2__/*r*_N_2__) over 3.7 nm Pd at different negative potentials in 20% N_2_/CO_2_ and 20% H_2_/CO_2_-saturated 1 M KHCO_3_ solutions.

We also measured the electrocatalytic reduction of CO_2_ without adding H_2_ to the feeds. As shown in Fig. 3, S2 and S3,† the current density becomes unstable at negatively shifted potentials, which is caused by poisoning from trace CO, a minor side product from the CO_2_ electroreduction.^16,35–37^ With the addition of H_2_, the stability of current density increases, indicating that the electrocatalytic reduction of CO_2_ is stabilized. X-ray diffraction (XRD) patterns, of 3.7 nm Pd under different atmosphere were obtained to investigate the active phase of Pd NPs, as shown in Fig. S4.† XRD pattern of 3.7 nm Pd under 20% N_2_/CO_2_ atmosphere matches well with that of Pd (JCPDS 46-1043), and the diffraction peak of the Pd (111) plane is located at 39.9°. When the atmosphere is switched to 20% H_2_/CO_2_, the diffraction peak of the Pd (111) plane quickly shifts to 38.8°, accompanied by a shift in all the other peaks. The pattern is consistent with that of PdH_0.706_ (JCPDS 18-0951), which is facilely generated under H_2_ atmosphere. Since the state and structure of the supported Pd NPs are not affected by water,^38^ the PdH_*x*_ active phase is expected to be stable in 20% H_2_/CO_2_-saturated 1 M KHCO_3_ solution.

Fig. 3c shows the electric charge ratios over 3.7 nm Pd at various potentials, calculated from the *I*–*t* plots, in Fig. 3a and b. At –0.1 V, the electric charge does not change over Pd NPs because surface PdH_*x*_ is stable and the rate of proton reduction can meet the requirement for the CO_2_ electroreduction. At –0.2 V, CO_2_ electroreduction is accelerated, and the rate of proton reduction cannot match the rate of CO_2_ electroreduction, resulting in a decrease of current density. The enhancement of the electric charge ratio is the highest at –0.3 V since the addition of H_2_ effectively stabilizes surface PdH_*x*_ that tends to decompose through hydrogen evolution. This enhancement of electric charge ratio indicates that the electrocatalysis is to some extent also promoted by H_2_ in the feeds, which has not been reported earlier.

The rate of formate production was enhanced when the reaction atmosphere was changed from 20% N_2_/CO_2_ to 20% H_2_/CO_2_. Fig. 3c shows the enhancement ratio of *r*_H_2__/*r*_N_2__ over 3.7 nm Pd at different negative potentials, where *r*_H_2__ and *r*_N_2__ represent the formate production rates in 20% H_2_/CO_2_ and 20% N_2_/CO_2_-saturated electrolyte solutions, respectively. The enhancement ratio of formate production rates is higher than that of electric charge accumulated in 1 h. For instance, *r*_H_2__ at –0.2 V reaches about 1.9 mol_formate_ mg_Pd_^–1^ h^–1^, and *r*_H_2__/*r*_N_2__ is more than twice that of *Q*_H_2__/*Q*_N_2__. The additional improvement is considered as a contribution from the thermocatalytic reaction,^39^ also identified in the isotope labeling experiment. The significant potential dependence of the *r*_H_2__/*r*_N_2__ and *Q*_H_2__/*Q*_N_2__ values over 3.7 nm Pd is attributed to the instability of Pd hydride and CO poisoning at more negative potentials during the electrocatalytic reduction of CO_2_. Similar enhancement effects over 2.4 nm and 7.8 nm Pd are shown in Fig. S2 and S3.† Since the strong hydrogen adsorption on small-sized Pd NPs could stabilize the surface Pd hydride, the current densities over 2.4 nm Pd were more stable than those over 3.7 nm Pd (Fig. 3 and S2†). Therefore, the *r*_H_2__/*r*_N_2__ and *Q*_H_2__/*Q*_N_2__ values over 2.4 nm Pd are smaller than those over 3.7 nm Pd. The lack of potential dependence of *r*_H_2__/*r*_N_2__ and *Q*_H_2__/*Q*_N_2__ over 7.8 nm Pd indicates that surface Pd hydride is difficult to form over large-sized Pd NPs, as also confirmed by the unstable current densities shown in Fig. S3.†

Based on the abovementioned results, thermocatalytic and electrocatalytic reduction of CO_2_ occur simultaneously as follows:^40–42^

(a) Thermocatalytic reduction reaction

3 Pd + *x*H_2_(g) ↔ H*

4 CO_2_(g) + H* ↔ HCOO*

5 HCOO* + H ↔ HCOOH*

6 HCOOH* ↔ HCOOH(l) + *

(b) Electrocatalytic reduction reaction

7 CO_2_(g) + * + e^–^ + H^+^(aq.) ↔ HCOO*

8 HCOO* + e^–^ + H^+^(aq.) ↔ HCOOH* + H_2_O(l)

9 HCOOH* ↔ HCOOH(l) + *

where * and H represent the palladium hydride active phase and the free H atom adsorbed on the catalyst surface, respectively. HCOO* and HCOOH* represent the corresponding intermediate species. It is clear from eqn (4) and (7) that the thermocatalytic and electrocatalytic reduction of CO_2_ share the same intermediate species HCOO*. Recent research on hydrazine electrooxidation indicates that the mechanistic origin of the EPOC effect lies in structurally similar activated transition states and/or adsorbed surface intermediates arising from hydrazine oxidation and decomposition.^14^ Therefore, the negative electrode potentials could affect and promote the heterogeneous catalytic reduction of CO_2_.

**Fig. 4:**
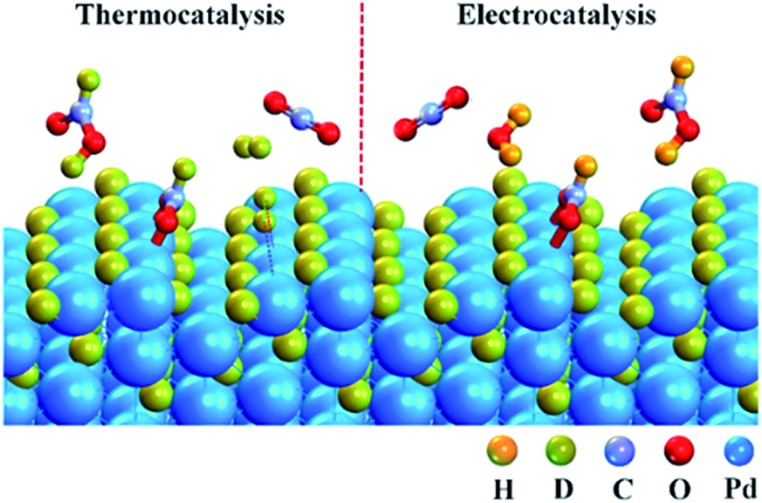
Schematic of heterogeneous thermocatalytic (left) and electrocatalytic (right) reduction of CO_2_ over Pd NPs conducted in a CO_2_ + D_2_ atmosphere.

Fig. 4 shows the schematic of CO_2_ + D_2_ reduction over Pd NPs. D_2_ is split into D atoms on the surface of Pd NPs, which subsequently diffuse into the Pd lattice to form the PdD_*x*_ phase. The atomic D on the PdD_*x*_ surface, derived from D_2_ dissociation, reacts with CO_2_ to form DCOO^–^ *via* the thermocatalytic pathway, whereas H^+^ in water reacts with CO_2_ to form HCOO^–^ *via* the electrocatalytic pathway. At –0.1 V and –0.2 V, the electrocatalytic reduction rate is constrained due to the low overpotentials and the percentage of HCOO^–^ is lower than that of DCOO^–^. Upon increasing the overpotential, the electrocatalytic reduction rate is accelerated, exceeding the thermocatalytic rate, and thus the electrocatalytic reduction pathway dominates for CO_2_ reduction at –0.4 V (Fig. 2).

**Fig. 5:**
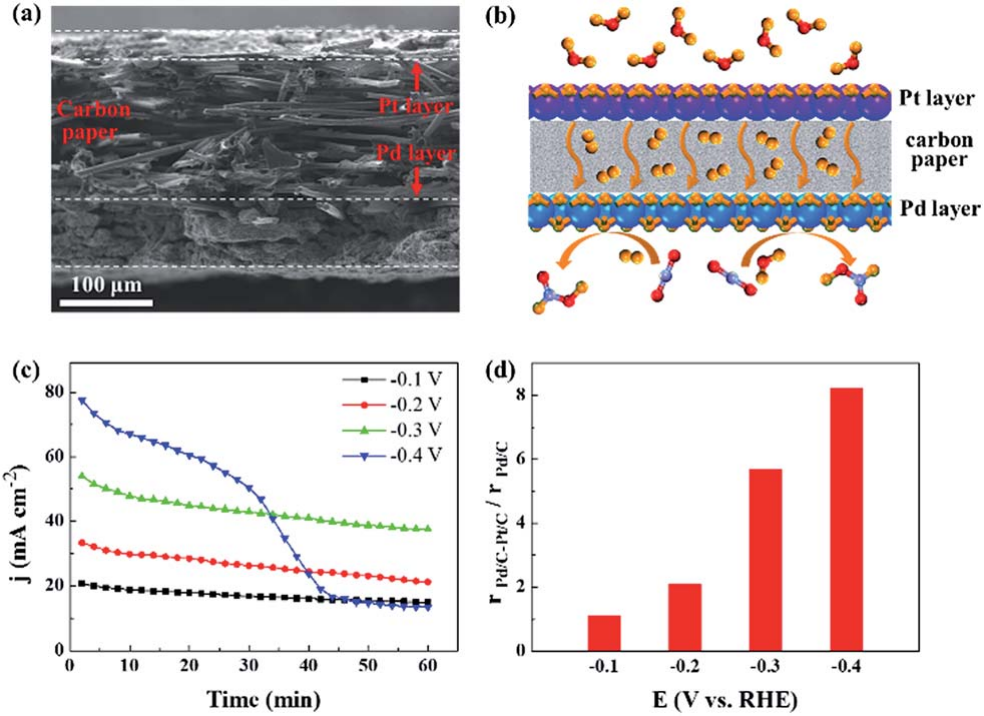
CO_2_ electroreduction over the Pd/C–Pt/C composite electrode in 20% N_2_/CO_2_-saturated 1 M KHCO_3_ solution. (a) Cross-sectional SEM image of the Pd/C–Pt/C composite electrode. (b) Schematic for the reaction mechanism in the Pd/C–Pt/C composite electrode. (c) Chronoamperometry curves of the Pd/C–Pt/C composite electrode at different negative potentials. (d) Rate enhancement ratio for formate production over the Pd/C–Pt/C composite electrode *versus* the Pd/C electrode at different negative potentials.

H_2_ generated from the electrolysis of water is promising for practical applications in CO_2_ reduction. Within the potential range for the CO_2_ reduction *via* CO_2_ + H_2_ reaction, the hydrogen evolution reaction can occur over Pt NPs, which would provide H_2_ by the electrolysis of water at the same negative potentials.^43,44^ We designed a composite electrode in which 3.7 nm Pd was deposited on the microporous layer, whereas commercial Pt/C catalyst was deposited on the other side of the carbon paper. The cross-sectional scanning electron microscopy (SEM) image and corresponding energy-dispersive X-ray spectroscopy mapping image of the electrode are shown in Fig. 5a and S5.† As illustrated in Fig. 5b, the Pt/C and Pd/C catalyst layers are separated by the carbon paper with a microporous layer, and H_2_ generated over Pt NPs diffuses across the carbon paper or the electrolyte solution towards the Pd NPs, which promotes the thermocatalytic and electrocatalytic reduction of CO_2_ over Pd NPs. The current density is clearly improved and stabilized after depositing Pt/C catalyst on the opposite side of the Pd/C catalyst layer (Fig. 3a and 5c). The activity and selectivity of the Pt/C electrode, used as a control in this study, were also measured, and only H_2_ was produced (Fig. S6†). The enhancement ratio for formate production with the Pd/C–Pt/C composite electrode is shown in Fig. 5d. The maximum value reaches 8.2 at –0.4 V as a result of the combination of thermocatalytic and electrocatalytic reduction of CO_2_.

## Conclusions

In summary, a significant EPOC effect was observed over Pd NPs during CO_2_ reduction to generate formate in 1 M KHCO_3_ solution at ambient temperature. Both thermocatalytic and electrocatalytic reduction of CO_2_ over Pd NPs were promoted by applying negative potentials and by adding H_2_ to the feeds, respectively. The shared reaction intermediate HCOO* over Pd NPs was proposed as the origin of the EPOC effect during the thermocatalytic and electrocatalytic reduction of CO_2_. Based on the abovementioned understanding, the Pd/C–Pt/C composite electrode was constructed for CO_2_ reduction without the direct addition of H_2_ to the feeds. H_2_ generated through water electrolysis over Pt NPs effectively promoted the formate production over Pd NPs. The significant rate enhancement ratio for CO_2_ reduction not only reveals a new example of EPOC in a low-temperature aqueous electrochemical reaction, but also provides an alternative strategy to promote the electrocatalytic reduction of CO_2_.
