# Correction: Electrochemical promotion of catalysis over Pd nanoparticles for CO_2_ reduction

**DOI:** 10.1039/c7sc90011b

**Published:** 2017-02-24

**Authors:** Fan Cai, Dunfeng Gao, Hu Zhou, Guoxiong Wang, Ting He, Huimin Gong, Shu Miao, Fan Yang, Jianguo Wang, Xinhe Bao

**Affiliations:** a State Key Laboratory of Catalysis , CAS Center for Excellence in Nanoscience , Dalian Institute of Chemical Physics , Chinese Academy of Sciences , 116023 , Dalian , China . Email: wanggx@dicp.ac.cn ; Email: xhbao@dicp.ac.cn; b College of Chemical Engineering , Zhejiang University of Technology , 310032 , Hangzhou , China; c University of Chinese Academy of Sciences , 100039 , Beijing , China

## Abstract

Correction for ‘Electrochemical promotion of catalysis over Pd nanoparticles for CO_2_ reduction’ by Fan Cai *et al.*, *Chem. Sci.*, 2017, DOI: 10.1039/c6sc04966d.



## 

Two minor mistakes were present in the published article and should be corrected as follows:

Page 2, eqn (2): in the denominator “total amount of Pd in electrode (in milligram)” should be revised to “total amount of Pd in electrode (in gram)”.

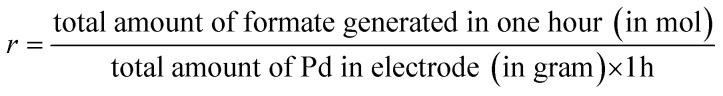



Page 3, column 2, paragraph 2, line 9: “…about 1.9 mol_formate_ mg_Pd_^–1^ h^–1^…” should be revised to “…about 1.9 mol_formate_ g_Pd_^–1^ h^–1^…”.

The Royal Society of Chemistry apologises for these errors and any consequent inconvenience to authors and readers.

