# Supplementary material for: Electrochemical promotion of catalysis over Pd nanoparticles for CO2 reduction
Source: Chem Sci. 2017 Jan 3;8(4):2569–73. doi: 10.1039/c6sc04966d (PMC5431665; doi:10.1039/c6sc04966d)
Supplement: Supplementary file 1 [file SC-008-C6SC04966D-s001.pdf]

Electronic Supplementary Material (ESI) for Chemical Science.
This journal is © The Royal Society of Chemistry 2017

Supplementary Information

Electrochemical promotion of catalysis over Pd nanoparticles for CO_2 reduction

Fan Cai,[a, c] Dunfeng Gao,[a] Hu Zhou,[b] Guoxiong Wang,[a*] Ting He,[a, c] Huimin Gong,[a] Shu Miao,[a] Fan Yang,[a] Jianguo Wang[b] and Xinhe Bao[a*]

[a]State Key Laboratory of Catalysis, CAS Center for Excellence in Nanoscience, Dalian Institute of Chemical Physics, Chinese Academy of Sciences, 116023, Dalian, China.

[b]College of Chemical Engineering, Zhejiang University of Technology, 310032, Hangzhou, China.

[c]University of Chinese Academy of Sciences, 100039, Beijing, China.

Corresponding authors:

wanggx@dicp.ac.cn (Guoxiong Wang); xhbao@dicp.ac.cn (Xinhe Bao)

Experimental section

Catalyst preparation

Carbon supported Pd NPs was synthesized using the method reported previously by our group, [29] the average particle size was controlled by varying the ratio of sodium citrate to palladium chloride and the reduction temperature. For the synthesizing of 2.4 nm Pd and 3.7 nm Pd, 0.5 mmol $PdCl_2$ (dissolved in 0.1 M HCl solution, Aldrich) was dissolved into 200 mL deionized water, and 4 mmol sodium citrate ([$Na_3C_6H_5O_7{\cdot}2H_2O$], Sinopharm Chemical Reagent Co. Ltd.) was added as a stabilizing agent, while 7.8 nm Pd was synthesized without the addition of sodium citrate. Then 212.8 mg Vulcan XC-72R carbon black (Carbot Corp.) was added, and sonicated for 30 min. After that, 25 mL of 0.1 mol L^{-1} $NaBH_4$ (Sinopharm Chemical Reagent Co. Ltd.) solution was added into the suspension to reduce Pd precursor under vigorous stirring at 25 °C for 3.7 nm Pd and 7.8 nm Pd, while the 2.4 nm Pd was reduced at 0 °C. After stirring for 6 h, the obtained Pd/C catalyst was filtered, washed and dried in a vacuum oven at 25 °C overnight. The nominal metal loading in Pd/C catalysts was 20 wt%.

Electrode fabrication

The catalyst ink containing Pd/C and Nafion ionomer was painted onto a piece of carbon paper (Toray TGP-H-060) coated with microporous layer and dried at 70 °C for more than 30 min to serve as porous electrode. The loading of Pd/C catalyst was 2.0±0.1 mg cm^{-2} and the Nafion content in the catalyst layer was 10 wt%. While the Pd/C-Pt/C composite electrode was prepared by painting commercial Pt/C (40 wt%, Tanaka Kikinzoku Kogyo K. K.) catalyst mixed with Nafion ionomer on the other side of the Pd/C electrode. The loading of Pt/C catalyst was 1.0±0.1 mg cm^{-2} and the Nafion content in the catalyst layer was 10 wt%.

Physicochemical characterization

Transmission electron microscopy (TEM) was carried out on a JEM-2100 microscope operated at 200 kV. Scanning Electron Microscopy (SEM) images and energy-

dispersive X-ray spectroscopy (EDS) mapping were performed on a JSM-7800F field emission scanning electron microscope. The cross-sectional sample was prepared by freezing the composite electrode in liquid nitrogen and breaking it off. The actual metal loadings of Pd/C catalysts were measured by a PerkinElmer ICP-OES (7300DV).

In-situ X-ray diffraction (XRD) patterns were collected with a Rigaku D/MAX 2500 diffractometer using a Cu Kα radiation (λ = 1.5418 Å) radiation source at 40 kV and 300 mA. Firstly, the Pd/C catalyst which was pressed into a tablet was purged by 20% N_2/CO_2, and the signals were collected continuously to obtain a stable XRD pattern. After that, the gas was switched to 20% H_2/CO_2 so that the XRD pattern changes under H_2 atmosphere could be detected.

CO_2 reduction experiments

CO_2 reduction experiments were carried out in an H-cell which was separated by Nafion 115 as reported in our previous work. [29] Pd/C electrode or Pd/C-Pt/C electrode (1 cm×2 cm), Ag/AgCl and Pt wire were used as the working electrode, reference electrode and counter electrode, respectively. The potentials were controlled by an Autolab potentiostat/galvanostat (PGSTAT 302N). All potentials in this study were measured against the Ag/AgCl reference electrode and converted to the RHE reference scale using E (vs. RHE) = E (vs. Ag/AgCl) + 0.21 V + 0.0591 × pH.

CO_2 reduction was performed in 1 M $KHCO_3$ solution which was saturated by 20% N_2/CO_2 or 20% H_2/CO_2. After 20% N_2/CO_2 or 20% H_2/CO_2 was purged into the 1 M $KHCO_3$ solution for 30 min to acquire corresponding saturated solution, controlled potential electrolysis was performed at each potential for 60 min. While the reduction performance at OCV was also measured in the same way without applying potential. The gas products of CO_2 electroreduction were detected by an *on-line* micro gas chromatography (GC) (Agilent 490) equipped with a TCD detector and Molsieve 5A column continuously. The liquid products were analyzed on a Bruke AVANCE III 400 MHz nuclear magnetic resonance (NMR) spectrometer.

Isotope labeling experiment was operated by replacing saturated gas from 20% H_2/CO_2 to 20% D_2/CO_2. $HCOO^-$ in the liquid products was quantified by ^{1}H-NMR spectrometry with water suppression by a pre-saturation method. Standard curve was made by using sodium formate ($HCOONa \cdot 2H_2O$, Sinopharm Chemical Reagent Co. Ltd.) which was dissolved in 1 M $KHCO_3$ with different concentrations as standard solutions and 1-Propanesulfonic acid 3-(trimethylsilyl) sodium salt (DSS, Sigma-Aldrich) as internal standard substance. The measurements of standard solutions or our electrolytes were conducted by mixing 0.5 mL sample with 0.1 mL of 6 mM DSS, and D_2O encapsulated into quartz capillary was added to lock field. While the $DCOO^-$ was quantified by ^{2}H-NMR spectrometry. The standard curve was made by using formic acid-D2 (DCOOD, J&K chemical) which was dissolved in 1 M $KHCO_3$ with different concentrations as standard solutions and dimethyl sulfoxide-D6 (DMSO-D6, Sigma) as internal standard substance. The measurements of standard solutions or our electrolytes were conducted by mixing 0.5 mL sample with 0.1 mL of 12.5 mM DMSO-D6, and D_2O encapsulated into quartz capillary was added to lock field. We also measured the amount of D_2O and HDO generated after constant-potential electrolysis at –0.2 V for 1 h by the ^{2}H-NMR spectrometry to evaluate the isotope exchange between adsorbed D and H^+. The measurements of the D_2O/HDO in the sample and water blank were similar to that of $DCOO^-$, except that the internal standard was changed to DSS-D4 (J&K chemical) and DMSO-D6 (Sigma) was used to lock filed.

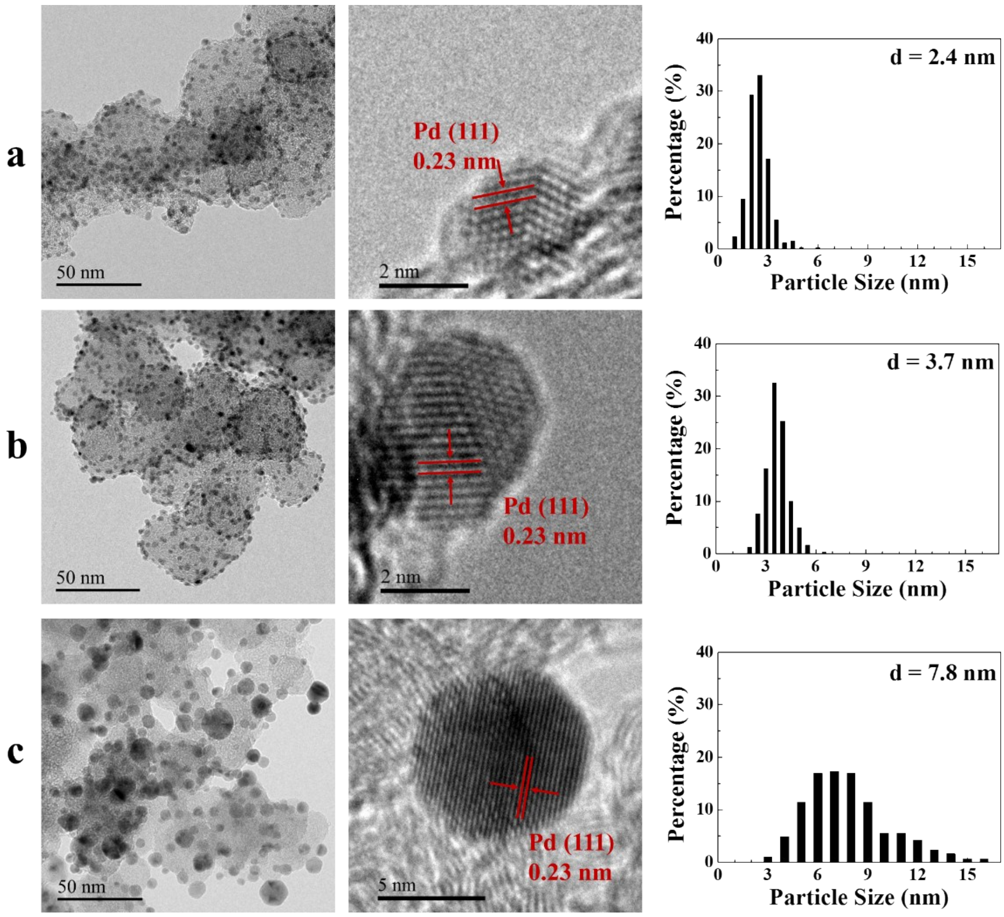

Figure S1 TEM and HRTEM images and corresponding histograms of (a) 2.4, (b) 3.7 and (c) 7.8 nm Pd. The histograms of particle size distribution are obtained by counting more than 300 particles for each from the TEM images.

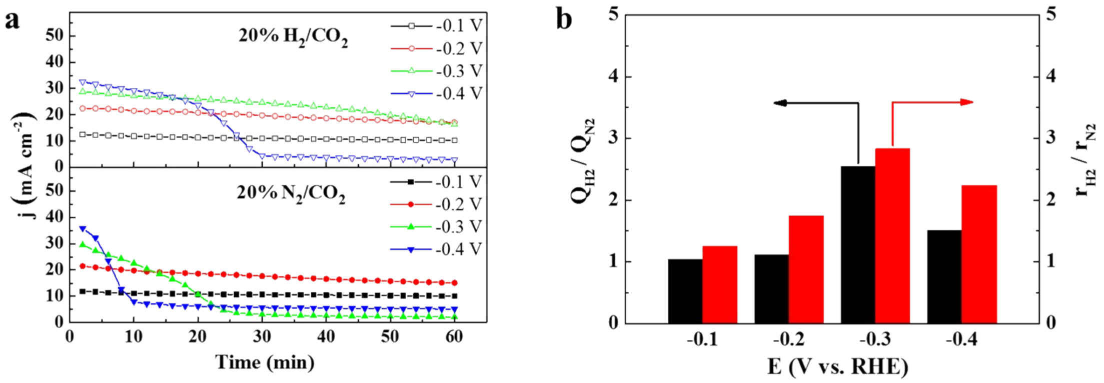

Figure S2 (a) Chronoamperometry curves of 2.4 nm Pd in 20% N_2/CO_2-saturated and 20% H_2/CO_2-saturated 1 M $KHCO_3$ solution. (b) Enhancement ratio of electric charge (Q_{H2}/Q_{N2}) and formate production rate (r_{H2}/r_{N2}) between 20% N_2/CO_2 and 20% H_2/CO_2-saturated 1 M $KHCO_3$ solution over 2.4 nm Pd at different negative potentials.

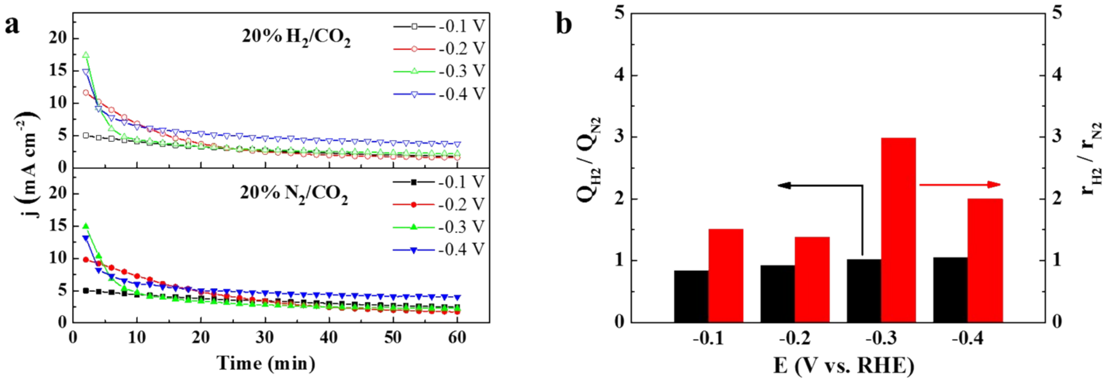

Figure S3 (a) Chronoamperometry curves of 7.8 nm Pd in 20% N_2/CO_2-saturated and 20% H_2/CO_2-saturated 1 M $KHCO_3$ solution. (b) Enhancement ratio of electric charge (Q_{H2}/Q_{N2}) and formate production rate (r_{H2}/r_{N2}) between 20% N_2/CO_2 and 20% H_2/CO_2-saturated 1 M $KHCO_3$ solution over 7.8 nm Pd at different negative potentials.

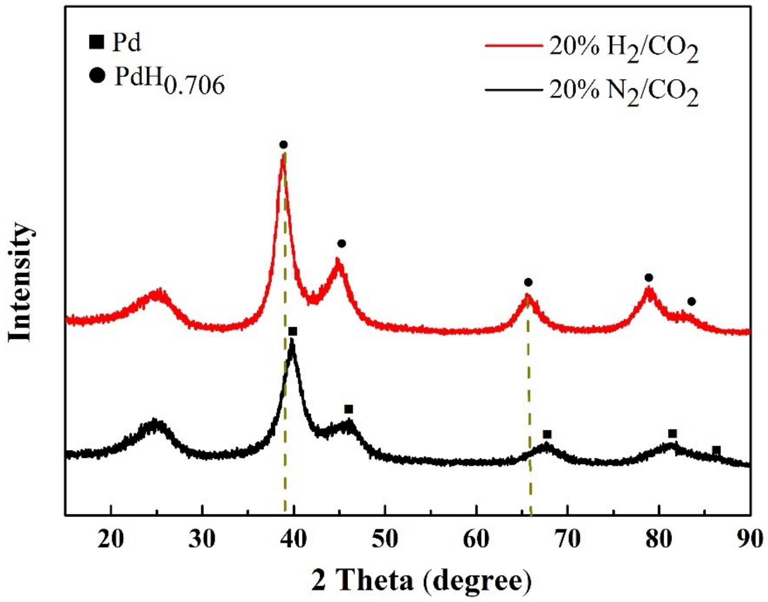

Figure S4 XRD patterns of 3.7 nm Pd in different atmospheres.

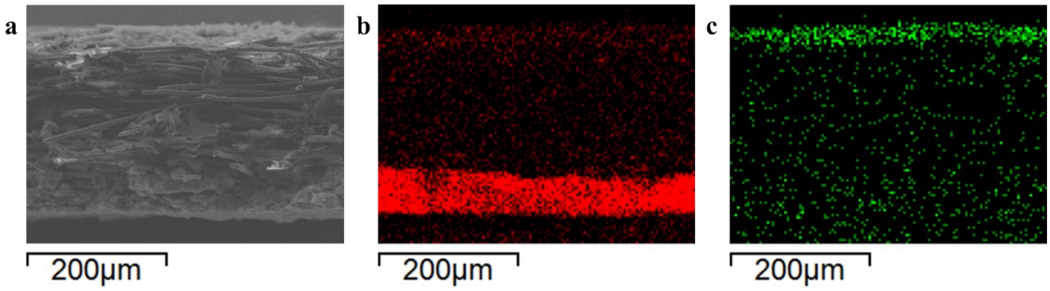

Figure S5 Cross-sectional SEM image (a) and EDS mappings of Pd/C-Pt/C composite electrode, (b) Pd signal: red and (c) Pt signal: green.

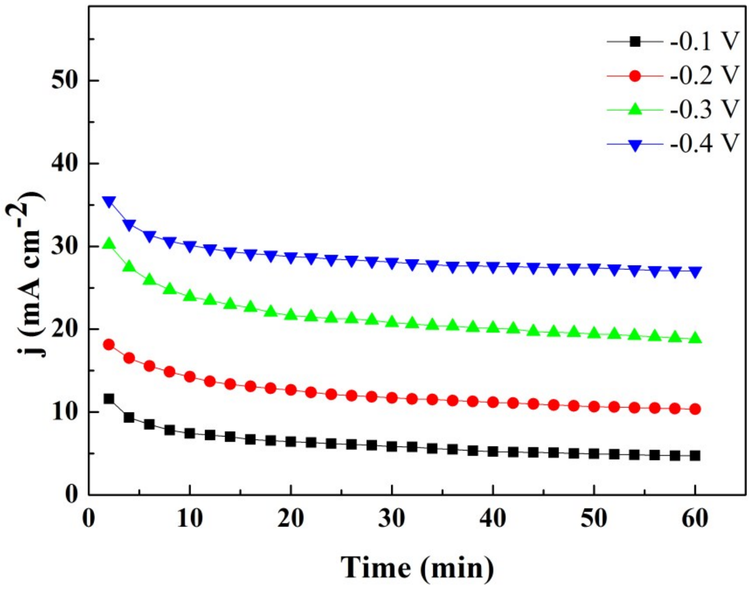

Figure S6 Chronoamperometry curves of Pt/C electrode in 20% N_2/CO_2 -saturated 1 M $KHCO_3$ solution.
